# Relationship of Common Vascular Anatomy to Cannulated Catheters

**DOI:** 10.1155/2017/5157914

**Published:** 2017-12-19

**Authors:** Paul Gagne, Karun Sharma

**Affiliations:** ^1^Vascular Breakthroughs, 85 Old Kings Highway North, Darien, CT 06820, USA; ^2^Interventional Radiology, Children's National Medical Center, 111 Michigan Ave. NW, Washington, DC 20010, USA

## Abstract

Superficial veins of the upper extremity are the primary location for placement of peripheral IV catheters (PIVC). It is believed that a significant portion of PIVCs placed may cross or abut valves and branching veins or occlude a significant portion of the vein, limiting the ability to aspirate blood from the PIVC. Two separate clinical investigations using ultrasound were performed to understand the potential interaction between PIVCs and the vein lumen and the venous valves and branches of the superficial veins of the upper extremity. One study with 35 adult volunteers interrogated 210 vein segments where a PIV would likely be placed. A second pediatric study evaluated 35 vein segments central to indwelling PIVCs. The combined data from the two studies showed that over 80% of adult veins and 85% of pediatric veins can properly accommodate 20-gauge and 22-gauge PIVC, respectively. Venous valves are frequent findings, either immediately peripheral to branching veins or at periodic 5 to 7 cm points. Antegrade blood flow can be restricted by a placed PIVC, while retrograde flow is very likely to be restricted by venous valves. Together, these findings may explain the difficulty in reliable aspiration of blood from PIVC.

## 1. Introduction

Superficial veins of the upper extremity are the primary location for placement of peripheral IV catheters (PIVC). Successful PIVCs have been defined as those that function to both infuse fluids and aspirate blood for the duration of a patient's need [1]. However, it is well known that PIVCs are often not able to aspirate blood for their intended dwell time. One study reported that 20% to 50% of PIVCs placed during surgery were unable to aspirate blood [2]. Other studies showed that 10–20% of freshly placed PIVC in volunteers were unable to aspirate blood [3–5]. Nonaspirating PIVCs have historically been ignored but a growing consensus, including the recent infusion nursing guidelines, suggests that PIVC-based blood collections in pediatrics and difficult access adult patients may reduce patient discomfort and improve care [6]. The reasons why PIVCs function for infusion but are unreliable for aspiration have not been explored, leaving clinicians unable to improve practices.

**Figure 1:**
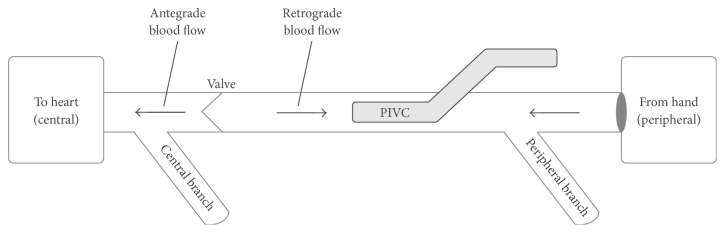
Blood flow through anatomy.

When PIVC-based aspiration is performed, the blood supply must come from the cannulated vein in which the catheter is placed with either peripheral to central flow around the catheter or retrograde flow to the catheter tip from a more central segment of the vein (Figure 1). Additionally, branches which merge with the target vein, either central or peripheral to the catheter, may contribute blood to refill the “aspiration reservoir” or target vein. Assuming a patent PIVC lumen, when aspiration fails there are two mechanisms of failure: (1) blood flow must be restricted or (2) the reservoir is empty peripheral to and/or central to the PIVC. We hypothesize that peripheral antegrade blood flow around a PIVC will be limited by the initial and, hence, residual, post-PIVC insertion, vein lumen size (i.e., residual cross-sectional area), whereas retrograde central blood flow may fail due to the presence of venous valves or the absence of branches which exist in the upper extremity superficial venous system. Given that the anatomy of veins, including venous valves and branches, likely changes from the peripheral hand to the antecubital fossa, PIVC placement location may also affect PIVC function. The details of this anatomy in the context of PIVCs have not been previously well studied.

To understand the potential interaction between PIVCs and the venous valves and branches of the superficial veins of the upper extremity, and specifically the hand and forearm where most PIVCs are placed, we performed an ultrasound-based study to evaluate the frequency of these anatomic features and their relationship to common cannulation sites. We further evaluated vein sizes to understand the hypothetical residual functional lumen that would result following a PIVC insertion. To broaden the applicability of findings, we first performed a study in adult patients and then adapted the study in pediatric patients to determine if findings in adults were similar in children. Our goal was to determine if the frequency and location of branches and valves as well as vein lumen size could explain the high failure rate of PIVC-based aspiration.

## 2. Materials and Methods

The adult investigation was a single-center study conducted at an outpatient treatment center. The study was reviewed and approved by the Western IRB. Thirty-five adult volunteers between the ages of 18 and 95 were recruited from the local population to participate. Upon informed consent, each patient was evaluated by a registered nurse with 5 years of experience in PIVC placement and use.

After visual exam, the nurse marked the most-likely PIVC insertion site on each patient's hand, forearm, and AC for both the left and right arm, a total of 6 markings using a tourniquet. The hand was defined as any insertion point peripheral to the wrist crease. The AC was defined as an insertion site 1 inch or less peripheral to the antecubital crease. The forearm was defined as any insertion central to the wrist crease and up to 1 inch peripheral to the AC. Ultrasound (US) imaging of each vein was performed by a registered vascular technician with 14 years of experience using a GE logiq S8 unit with an 18 mhz high frequency linear “hockey stick” probe. The patient was seated upright with the arm horizontal out from the body, without a tourniquet. No PIVC was actually placed, but the position of the catheter tip if the IV was placed at the marked insertion site was used as the reference point for the valves and branches. The hypothetical PIVC catheter length was 1 inch.

**Figure 2:**
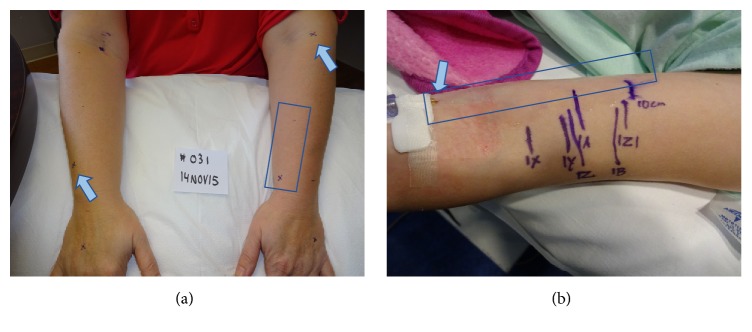
(a) Adult study patient with marked hypothetical insertion sites (arrow) and interrogated segments (box) and (b) pediatric study patient with PIV (arrow) and marked ultrasound findings in 10 cm segment (box).

**Figure 3:**
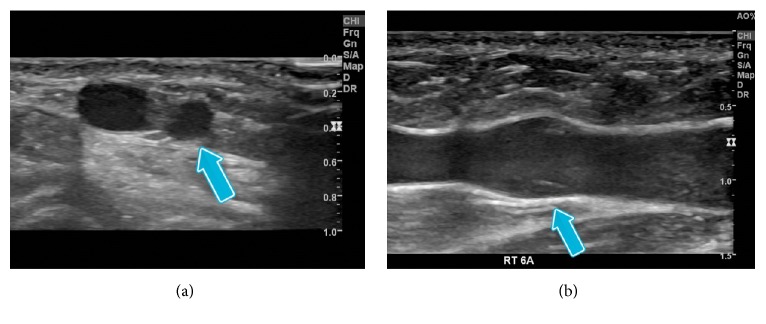
(a) Ultrasound image of branching vein (arrow) joining studied vein and (b) ultrasound image of valve showing two linear and symmetrical valve leaflets in dilated vein sinus (arrow).

Resting target vein diameter and vein depth from the skin were measured at the marked insertion point. Recognizing that the effect of blood flow through a vein branch or valve remote to the catheter insertion site or tip might affect the ability to aspirate blood through the catheter, we also evaluated the pertinent venous structures central to the theoretical catheter insertion sites of the target vein. Using a ruler, an end-point 10 cm from each insertion point was arbitrarily selected and marked to denote the furthest distance evaluated with US in each vein (Figure 2(a)). The location of each valve and branch-point along the 10 cm segment of vein was noted as a distance from the insertion site. A valve was defined as a horizontal or angular linear echogenic structure inside the vein lumen often in continuity with the dilated segment of a vein sinus (Figure 3). A branch was defined as an ultrasound visualized lumen that merged with the axial or target superficial vein (Figure 3(b)) being interrogated based on the initial insertion site marking.

The pediatric investigation was performed subsequent to the adult study and evaluated venous anatomy central to indwelling PIVCs. Pediatric subjects were enrolled at a large tertiary care children's hospital. The study was approved by the local institutional review board. This cohort consisted of 35 children with indwelling peripheral IVs who were referred for placement of a peripherally inserted central catheter (PICC). During routine ultrasound evaluation for PICC placement, interrogation of the superficial vein anatomy at the existing PIVC insertion site was also performed. The ultrasound imaging was performed using a Philips Epiq ultrasound unit with a 15 MHz hockey stick vascular access probe. Imaging was performed by a fellowship trained interventional radiologist as the patients lay supine with the arm in extension and without the use of a tourniquet.

Similar to the adult study, each vein was assessed for diameter and depth from the skin. Only one actual PIVC location was interrogated per patient rather than the six hypothetical locations interrogated in the adult study. The gauge size of the PIVC (22 G or 24 G) was noted, and the size and depth of the vein were measured at mid PIV catheter rather than a hypothetical insertion site. The location of valves and branching veins was recorded as described above for the 10 cm vein segment central to the catheter insertion point (Figure 2(b)).

## 3. Results

### 3.1. Adult Cohort Results

The adult ultrasound study evaluated 35 subjects, 17 females, with an average age of 52 years (range 18–89), and an average BMI of 25.7 (range 18–39). Within these 35 patients, a total of 210 upper extremity superficial vein segments equally divided between the hand, forearm, and AC were evaluated.

Veins of the hands were the smallest on average followed by the forearm and AC. The smallest veins (<=1 mm diameter) were exclusively found in the hand, whereas large veins (>=6 mm) were exclusively in the AC. Veins 2-3 mm in width were present in all three locations (Table 1). There was no correlation in the diameter of the veins in the hand, forearm, or AC. Patient age and BMI were not correlated to vein diameters, whereas gender was correlated. Female veins were smaller on average, with significant difference in the forearm and hand (hand: 1.8 versus 2.3 mm *p* < 0.01; forearm: 2.4 versus 3.4 mm *p* < 0.01; AC: 4.1 versus 4.6 mm *p* = 0.14).

Over 10% of all imaged segments in adults had no branching veins present. Patient age, gender, and BMI were not found to be statistically significant predictors of vein segments without branches. AC veins were least likely to have branching veins within the evaluated segment. On average, a branch could be found every 5–7 cm in the target vein segments evaluated.

In 19% of observed adult veins, the branching vein had a larger diameter than the studied vein, possibly suggesting that the branching vein was the dominant source of peripheral to central blood flow in the superficial venous system rather than the studied vein. Although we attempted to determine blood flow direction in the studied “target” and branching veins, spontaneous flow velocity was too low for reliable Doppler US assessment.

Over 26% of all imaged segments in adults had no valves identified. Patient age, gender, and BMI were not found to be statistically significant in differentiating segments with and without valves. Veins of the antecubital fossa were much less likely to have valves, whereas veins of the hand and forearm were nearly equal in the frequency of valves identified. In those vein segments of the hand with valves, there were often multiple valves identified within the 10 cm segment studied (Table 2).

**Figure 4:**
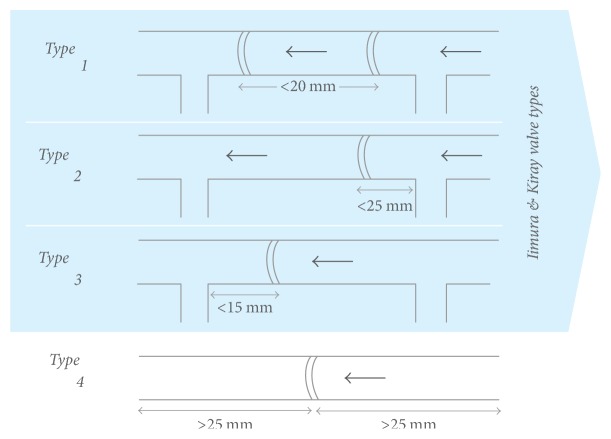
Modification of Kiray and Iimura's classification system. Type 1 valve - within 20 mm of another central valve, with no intermediate branches; Type 2 valve - between two branches, closer to the peripheral branch, and within 25 mm of that peripheral branch.; Type 3 valve - between two branches, closer to the central branch, and within 15 mm of that central branch; Type 4 valve was defined as having no branches or other valves within (+/−) 25 mm of the valve.

Analysis of the relation between individual branches and valves and the target vein segments revealed that the predominant configuration was a valve immediately peripheral to a branch or type 3 (Table 3). In addition to type 3 valves, type 1 and type 4 or 80% of all valves would be restrictive of retrograde blood flow (Table 3, Figure 4).

### 3.2. Pediatric Cohort Results

The pediatric study evaluated 35 subjects (21 female) with a mean age of 11.7 (range 5–15) years and a BMI of 19 kg/m2 (range 14–26). Peripheral veins with existing PIVs were evaluated to assess branch and valve frequency. This ultrasound evaluation in the pediatric cohort was more challenging and potentially less reliable because nearly 50% of the studied segments had portions, typically immediately central to the PIV, which were collapsed or had an extremely narrowed lumen over a distance of 1 to 5 cm.

Vein diameter in the cannulated segment of the pediatric cohort was similar to adults in the hand, but smaller in the forearm and AC by approximately 1 mm. Both groups showed similar standard deviation in size (Table 1). In terms of branch and valve frequency, 33% of pediatric veins, in general, had no branches seen over the evaluated 10 cm segment. Of the assessed hand segments, 60% had no identifiable branches and another 30% had only one identifiable branch. More than 57% of the pediatric veins had no valves identified in the studied segment. These children with PIVCs exhibited valves in only 1 of 3 assessed AC segments and 3 of 15 assessed hand segments. Due to the limited pediatric valve and branch data, a comparison to the adult data was not meaningful (Table 2).

The relationship between individual branches and valves showed that the predominant vein configuration was of a valve immediately peripheral to a branch. Similarly, 80% of valves were restrictive of reverse (central to peripheral) blood flow; however, the percentage of valves with no nearby valves or branches was much higher (Table 3, Figure 4).

### 3.3. Discussion

The relationships between PIVC gauge and vein size and between PIVC placement and venous branches and valves are likely factors in the ability to aspirate blood from a PIVC. The cross-sectional area of the vein determines the percentage of the lumen occluded by the PIVC and therefore the volume of blood able to flow around the PIVC [7]. Nearby centrally located branching veins represent potential sources of blood flow to the catheter. Valves, alternatively, impede retrograde blood flow and may contribute to the collapse of the vein deprived of retrograde filling around the catheter or, in some cases, cover and occlude the end of the catheter itself. Together, the combination of a relatively small vein around a PIVC, branches remote from the PIVC, and valves central to the PIVC tip may create a segment with limited blood flow availability and a nonexistent “aspiration reservoir” and may explain the variable success with PIVC aspiration that is observed clinically (Figure 3). The aim of the two studies discussed herein was to describe the venous anatomy found in upper extremity PIVC placement locations by documenting vein size, as well as branch vein and valve frequency and location in order to assess the likelihood of limited blood flow segments.

PIVC failure is an understudied phenomenon. While previous literature (cross reference) has noted that PIVCs are unreliable for blood aspiration, this is typically a secondary finding in many studies examining PIVC infusion properties. One study that attempted to define the root cause of unreliable aspiration showed that likelihood of ability to aspirate was related to gauge size of the placed PIVC [2]. This relationship was nonlinear, with 18-gauge PIVs being more reliable than both 16- and 20-gauge PIVs. Two other studies assessed the frequency of venous valves [8, 9], but data was limited to the major basilic and cephalic veins and used cadaveric specimens which limits a comprehensive dynamic understanding of vein function that is available in patients. The remaining literature on PIVC success rates has focused on practitioner skill and PIV characteristics rather than patient venous anatomy [10, 11]. The data gathered herein focuses on vein anatomy as a critical factor in PIVC function.

It is generally accepted that, for veins to maintain adequate blood flow around a partially occlusive indwelling catheter, the vein should be larger than twice the diameter of the catheter [7] Using this metric, our data suggests that adult peripheral veins are likely to be large enough to accommodate 20- and 22-gauge PIVCs, but an 18-gauge catheter may occlude too much of a vein's lumen for antegrade flow around the catheter (Table 1). Similarly, the veins of children can theoretically accommodate 24- and 22-gauge PIVC but a 20-gauge catheter may occlude too much lumen for aspiration of blood flowing antegrade. However, this is not equal across genders. Only 50% of studied adult female hand veins could accommodate a 20 G PIVC versus 86% in men. Without adequately maintained blood flow around the PIVC, the vein lumen may experience slower flow and increased clotting which also limits antegrade venous flow available during aspiration.

During aspiration from a PIVC, the vein and blood flow are under negative pressure. Retrograde blood flow from branching veins central to the PIVC tip could hypothetically provide adequate blood for continuous aspiration in supplementing any antegrade flow around the PIVC. However, the ability of more central branches to provide blood flow is limited by the proximity of the PIVC tip to that branch. Our data show that vein branches are seen approximately every 5–7 cm along a cannulated vein in adults. Assuming random placement in a segment, the next branch central to the PIVC tip could be up to 6 cm away and on average will be 3 cm central to the catheter tip. Given that veins are pliant, negative pressure during aspiration may cause the vein lumen to collapse if the blood flow available for aspiration is less than the suction pressure. This collapse may be less likely if the point of aspiration is close to a branch because of the increased blood flow available and theoretically because of the change in vein wall structure around the intersection point of two veins.

Finally, the frequency of valves preventing retrograde flow and the relationship between those valves and branches central to the PIVC also likely affects PIVC aspiration success. Similar to the previous literature [8, 9] and conventional teaching, the adult and pediatric ultrasound data documented twice as many instances of valves peripheral to a branch as instances of branches peripheral to a valve (Table 3). Our ultrasound data additionally identified 20% of valves in arm veins as having no branches or other valves within 25 mm either peripheral or central. Though stand-alone valves were not documented in previous literature, this is a known valve configuration in the leg veins where higher valve frequency is required to prevent retrograde flow caused by gravity.

**Figure 5:**
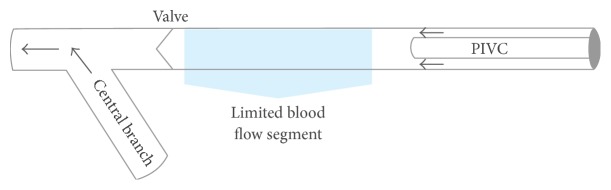
Diagram of hypothetical limited flow segment created by the confluence of vein size, distant central branch, and valve between branch and catheter.

In sum, many veins are large enough to maintain an adequate lumen around a placed PIVC; however, this is not equal across genders and cannulation sites. Further, while venous branches are relatively frequent and likely to be only a few several centimeters from the PIVC tip, intervening valves that prevent retrograde flow are commonly found between the PIVC tip and the central branch. These combined findings suggest that short segments of vein central to a PIVC tip in which there is limited to no spontaneous blood flow (Figure 5) are common and this may explain the variable success with PIVC aspiration that is observed clinically.

Our data suggest that, in order to optimize PIVC success, veins would be cannulated such that the tip of the PIVC is placed close to a central branch while avoiding intermediate valves. Furthermore, there is an optimal PIVC gauge for each vein balancing the vein size and the catheter lumen size. And finally, ultrasound visualization of vein size and local anatomy during insertion may increase the likelihood of optimal PIVC placement and the ability of placed PIVC to maintain full function for the duration of its need.

A limitation of the results is that the data combines findings from two separate clinical protocols. While over two hundred peripheral vein segments were imaged in the adult study, the pediatric study was limited by the availability of pediatric patients with indwelling PIVCs. Secondly, the adult interrogated likely PIVC placement locations, whereas the pediatric study interrogated vein segments around existing PIVC. Unfortunately, the data collection available from the pediatric vein segments was hindered by the smaller anatomical size and superimposed changes to the vessel potentially from the presence of the PIVC.

## 4. Conclusions

PIVCs placed in the superficial veins of the upper extremity are known to lose the ability to aspirate blood prior to the intended dwell time. As an alternative to repeated venipuncture, PIVC-based blood collections are desired but their unpredictability leaves them underutilized. The relationship between the venous anatomy immediately peripheral to and central to a placed PIVC had not been previously well researched as a potential cause of the limited ability to aspirate. The two clinical studies herein documented that the relationship between vein diameter and catheter diameter has the potential to limit blood flow around the PIVC in a significant proportion of veins. Furthermore, the distance between a PIVC and a more central branch is likely to contain a venous valve preventing retrograde blood flow. Therefore, occlusion of the vein by the PIVC, a long distance to the merging branches, and impeding venous valves may all play a role in the inability to aspirate blood from a PIVC.

Further research of indwelling PIVCs is required to validate the hypothesized blood flow restrictions around PIVC, as well as assessing the degree to which the various venous anatomic factors affect the ability to perform PIVC-based aspiration before the findings can be used to guide changes in clinical practice.

## Figures and Tables

**Table 1:** Vein sizes by location in adult and pediatric patients.

Adult findings	Vein diameter avg	% of veins with diameter 1.5x a *standard 20-gauge* PIV (>1.62 mm)
Hand (*n* = 70)	2.0 sd 0.7	71%
Forearm (*n* = 70)	2.9 sd 1.0	93%
AC (*n* = 70)	4.4 sd 1.8	97%

Pediatric findings	Vein diameter avg	% of veins with diameter 1.5x *observed* PIV (50% 22 G, 50% 24 G)

Hand (*n* = 14)	1.7 sd 0.8	86%
Forearm (*n* = 18)	1.9 sd 0.6	94%
AC (*n* = 3)	3.4 sd 1.5	100%

sd = standard deviation.

**Table 2:** Frequencies of branches and valves in adults^*∗*^.

Location	Avg frequency of branches in adults	Avg frequency of valves in adults
Hand	1 per 5 cm	1 per 6 cm
Forearm	1 per 6 cm	1 per 7 cm
AC	1 per 7 cm	1 per 17 cm

^*∗*^Pediatric data excluded due to low frequency of branches observed.

**Table 3:** Comparison of valve patterns across studied populations.

Valve pattern	Adult ultrasound (blended all veins)	Pediatric ultrasound (blended all veins)
Type 1 (valve next to valve)	6.7%	0%
Type 2 (valve central to branch)	19.2%	20%
Type 3 (valve peripheral to branch)	54.2%	40%
Type 4 (valve alone)	20.0%	40%
